# Validation of the Pittsburgh sleep quality index in community dwelling Ethiopian adults

**DOI:** 10.1186/s12955-017-0637-5

**Published:** 2017-03-27

**Authors:** Mohammed Salahuddin, Tarekegn Tesfaye Maru, Abera Kumalo, Seithikurippu R. Pandi-Perumal, Ahmed S. Bahammam, Md Dilshad Manzar

**Affiliations:** 1 Department of Pharmacy, College of Health Sciences, Mizan-Tepi University, Mizan Aman, Ethiopia; 2 Department of Biomedical Sciences, College of Health Sciences, Mizan-Tepi University (Mizan Campus), Mizan Aman, Ethiopia; 3 Somnogen Canada Inc, College Street, Toronto, ON Canada; 4 The University Sleep Disorders Center, King Saud University, Riyadh, Saudi Arabia; 5 National Plan for Science and Technology, College of Medicine, King Saud University, Riyadh, Saudi Arabia

**Keywords:** PSQI, Catha edulis, Ethiopia, Insomnia, Pittsburgh sleep quality index, Sleep, Substance abuse

## Abstract

**Background:**

The applicability of the Pittsburgh Sleep Quality Index (PSQI) in screening of insomnia is demonstrated in various populations. But, the tool has not been validated in a sample of Ethiopians. Therefore, this study aimed to assess its psychometric properties in community dwelling Ethiopian adults.

**Material and methods:**

Participants (*n* = 311, age = 25.5 ± 6.0 years and body mass index = 22.1 ± 2.3 kg/m^2^) from Mizan-Aman town, Southwest Ethiopia completed the PSQI and a semi-structured questionnaire for socio-demographics. Clinical interview for screening of insomnia according to the International Classification of Sleep Disorders was carried out as a concurrent validation measure.

**Results:**

Overall, the PSQI scale did not have floor effect and ceiling effects. Moderate internal consistency (Cronbach’s alpha was 0.59) and sufficient internal homogeneity as indicated by correlation coefficient between component scores and the global PSQI score was found. The PSQI was of good value for screening insomnia with optimal cut-off scores of 5.5 (sensitivity 82%, specificity 56.2%) and the area under the curve, 0.78 (*p* < 0.0001). The PSQI has unidimensional factor structure in the Ethiopian community adults for screening insomnia.

**Conclusion:**

The PSQI has good psychometric validity in screening for insomnia among Ethiopians adults.

**Electronic supplementary material:**

The online version of this article (doi:10.1186/s12955-017-0637-5) contains supplementary material, which is available to authorized users.

## Introduction

Sleep disorders are becoming endemic in both developed and developing societies [1–4]. More than half of the population across the globe is affected by some type of sleep disturbances and about one fifth of adults have chronic sleep disorder [1–5]. Sleep problems are implicated in poor health conditions characterized by impaired social relationships, neurologic, and/or psychiatric conditions, drowsy driving, risk-taking behavior, occupational accidents, and heightened risk of cardiovascular events [3–5]. Majority of the Ethiopian university students are sleep disturbed, which adversely affected their psychological health [2, 3].

Insomnia and its subjective symptoms e.g. difficulty initiating sleep, short sleep duration and poor sleep quality are the main dimensions of poor sleep in Ethiopia [2, 3]. Ethiopia is a developing country of Africa with limited trained sleep health professionals. There is no indigenously developed questionnaire tool for the assessment of sleep quality. Additionally, no work has been done to validate known tools that assess sleep quality in Ethiopians. In such circumstances, the availability of a validated questionnaire tool to assess sleep health is necessary. There are more than 80 different languages in the country with majority of the Ethiopians having low to high level of proficiency of spoken Amharic, which is the national language. However, the reading proficiency is more variable because of differences in script for major groups of languages.

The Pittsburgh Sleep Quality Index (PSQI) is the most widely used instrument in diagnosis of sleep disorders including insomnia in different populations [5–9]. The questionnaire has twenty-four items of which nineteen self-reported items are added non-linearly to generate seven components. The scores of these components are pooled linearly to get the global PSQI score, which is a measure of sleep health for the period of the 1 month immediately preceding the time of administration. The tool is easy to understand, patient compliant and requires about 5 min to be completed. The validity of the PSQI is well established in various clinical and non-clinical populations, people of different regions and ethnicities [5, 6, 10]. However, few studies have investigated the psychometric properties of the PSQI in African population. It has never been validated in Ethiopian populations [5, 7]. The present study therefore sought to validate the PSQI in a sample of community dwelling Ethiopian adults.

## Material and methods

Households were selected by simple random sampling (SRS) method across Mizan-Aman town, Bench Maji Zone, Southwest, Ethiopia. Further, only one adult member (chosen randomly) from every selected house was interviewed. Three hundred and eleven out of an initial 550 adults who were screened and who had been found qualified were administered the survey and fully completed it. Ethiopia is known for cultivation of Chat-a psycho-stimulant and coffee. The consumption of chat and/or coffee is highly prevalent in the Ethiopian community. Chat has been reported to be associated with poor sleep [2, 3]. The previous African study reporting validation of the PSQI on college students in Nigeria did not report about Chat habits [7]. Moreover, the study only involved college students [7], whereas our study assessed the validity of the PSQI in the community dwelling adults. The mean age and body mass index (BMI) of the participants were 25.5 ± 6.0 years and 22.1 ± 2.3 kg/m^2^, respectively. Exclusion criteria consisted of self-reported problems with memory. A detailed explanation regarding the purpose and procedures of the study was given to the volunteers. Even though, Amharic is the most widely spoken language but its reading proficiency level is limited. Therefore, instructor administered original English version of the PSQI was employed. Semi-structured tool for demographics and the PSQI were employed. All the participants were interviewed by an experienced sleep researcher blinded to the PSQI score for the presence of insomnia according to the International Classification of Sleep Disorders, revised criteria (ICSD-R). These criteria included: (i) Almost nightly insufficient amount of sleep, (ii) Not feeling rested after usual sleep and (iii) Mild to severe impairment of social or occupational functioning, (iv) Complaint of restlessness, irritability, anxiety, daytime fatigue, and tiredness. The subjects were screened as insomniacs if they had either (i or ii) of the condition and at least mild complaints related to both (iii) and (iv) [7]. The PSQI measures sleep quality for the month period just preceding the interview. But, it has been found to be a valid and reliable measure of insomnia in some populations [5, 7–9].

### Statistical analysis

The statistical package SPSS version 16.0 (SPSS Inc., Chicago, USA) was used. The PSQI is composed of nineteen self-reported items. The scores of these individual items are added non-linearly to get seven component scores. These components are measured variables and should not be confused with component/factor term (a latent variable) used in factor analysis [6, 11]. The Cronbach alpha test was used to assess internal consistency. Internal homogeneity was tested by correlation analysis between PSQI components and the global scores. Discriminative validity was assessed by test of difference; Mann Whitney for the structured categorical variables (the PSQI components) and *t*-test for the global PSQI score. The diagnostic validation was performed by receiver operating curve (ROC) analysis. The screening by the sleep expert based on clinical interview served as the gold standard and the global PSQI score was the test variable. Sensitivity, specificity, area under the curve (AUC), and cut off score were assessed. Exploratory factor analysis (EFA) was performed using principal component analysis for initial estimate. This was followed by maximum likelihood estimation with direct oblimin rotation. EFA investigated two types of the PSQI i.e. one with all seven components and other with five components (sans the PSQI components of medicine use and daytime dysfunction). Confirmatory factor analysis (CFA) was performed using maximum likelihood. A value of up to 0.05 indicated good fit for both root mean square residual (RMR) as well as root mean square error of approximation (RMSEA). A value of more than/equal to 0.90 indicated good fit for both goodness of fit index (GFI) as well as adjusted goodness of fit index (AGFI) [12]. Lesser value of expected cross-validation index (ECVI) indicates better fit- employed as a relative measure of fit. A comparative fit index (CFI) of no less than 0.95, and *χ* ^2^/df ratio of less than 3 indicated an acceptable fit between a model and the data [13].

## Results

The socio-demographics of the community dwelling Ethiopian adults participating in the study are given in Table 1. Table 2 shows the item analysis (i.e. component analysis in this case) of the PSQI in the study population. The mean global PSQI score was 7.0. Majority of the participants reported habit of tea/coffee (99.0%), alcohol intake (74%) and Chat chewing (52.1%), (Table 1).

**Table 1:** Socio-demographics of community dwelling Ethiopian adults

Characteristics	Mean ± SD/Frequency
Age (yr)	25.45 ± 5.99
BMI (Kg/m^2^)	22.07 ± 2.30
Gender	
Male	276 (88.7%)
Female	35 (11.3%)
Ethnicity	
Bench	87 (28%)
Kaffa	75 (24.1%)
Oromo	38 (12.2%)
Amhara	40 (12.9%)
Tigre	7 (2.3%)
Others	64 (20.6%)
Education	
Illiterate	1 (0.3%)
Can read and write	99 (31.8%)
Primary (1–8)	21 (6.8%)
Secondary (9–12)	76 (24.4%)
College/University	114 (36.7%)
Occupation	
Farmer	36 (11.6%)
Government Employee	34 (10.9%)
Merchants	17 (5.5%)
Housewife	1 (0.3%)
Others	223 (71.7%)
Religion	
Orthodox Christian	162 (52.1%)
Protestants Christian	101 (32.5%)
Islam	44 (14.1%)
Others	4 (1.3%)
Monthly Family Income (In Birr)	
Very Low (less than 445)	15 (4.8%)
Low (446–1200)	186 (59.8%)
Average (1201–2500)	87 (28.0%)
Above average (2501–3500)	16 (5.1%)
High (greater than 3500)	7 (2.3%)
Parents	
Single	210 (67.5%)
Married	98 (31.5%)
Divorced	3 (1.0%)
Sleep	
Global PSQI score	6.96 ± 3.34
ICSD-R Classification	
Insomniac/normal	206 (66.2%)/105 (33.8%)
Substance use/Habits	
Chat user/non-user	162 (52.1%)/149 (47.9%)
Alcoholic/non-alcoholic	230 (74%)/81 (26%)
Smoker/non-smoker	79 (25.4%)/232 (74.6%)
Tea/Coffee consumer/non-consumer	308 (99.0%)/3 (1.0%)
Beverage consumer/beverage non-consumer	129 (41.5%)/182 (58.5%)

*BMI* body mass index, *PSQI* Pittsburgh sleep quality index; *ICSD-R* international classification of sleep disorders, revised criteria

**Table 2:** The distribution of the Pittsburgh Sleep Quality Index (PSQI) scores in community dwelling Ethiopian adults

Components of the PSQI	PSQI sub-component	Frequency	Percentage
PSQI component of sleep duration	≥7 h	95	30.5
	6–7 h	53	17.0
	5–6 h	28	9.0
	<5 h	135	43.4
PSQI component of sleep disturbances	0	47	15.1
	1	261	83.9
	2	3	1.0
	3	0	0
PSQI component of sleep latency	0	36	11.6
	1	113	36.3
	2	124	39.9
	3	38	12.2
PSQI component of daytime dysfunction	0	293	94.2
	1	14	4.5
	2	4	1.3
	3	0	0
PSQI component of sleep efficiency	>85%	105	33.8
	75–84%	24	7.7
	65–74%	27	8.7
	<65%	155	49.8
PSQI component of sleep quality	Very good	97	31.2
	Fairly good	126	40.5
	Fairly bad	54	17.4
	Very bad	34	10.9
PSQI component of sleep medication	Not during the past month	306	98.4
	Less than once a week	3	1.0
	Once or twice a week	2	.6
	Three or more times a week	0	00

Ceiling or floor effects were considered to be present if more than 15% of respondents achieved the highest or lowest score, respectively [14, 15]. Overall, the global PSQI score did not have floor and ceiling effects; 5.1% of Ethiopian adults reported a minimum score of zero, and none reported a maximum score of 21, respectively. However, individual components of the tool did show floor and ceiling effects. All the PSQI components except for sleep latency showed floor effect i.e. more than 15% of respondents achieved the lowest score [14, 15]. Nevertheless, ceiling effect was observed only for components of sleep duration and sleep efficiency i.e. more than 15% of respondents achieved the highest score [14, 15].

**Fig. 1:**
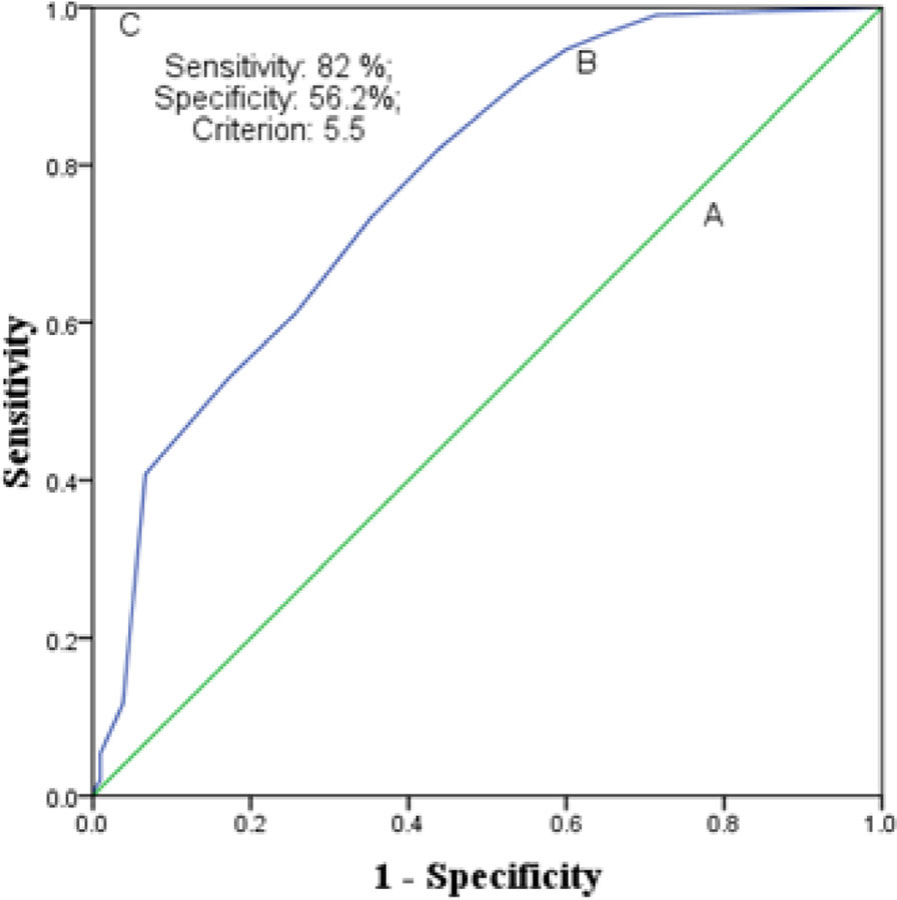
Receiver operator curves (*A*) No discrimination (AUC = 0.5) (*B*) Experimental test (0.78 (*p* < 0.001)) and (*C*) Perfect test (AUC = 1.0) in community dwelling Ethiopian adults

The internal consistency test of the PSQI scores showed a Cronbach’s alpha of 0.59, a value suggesting moderate consistency. Cronbach’s alpha increased by 0.03 (from 0.59 to 0.62) on removing the PSQI components of medicine use and daytime dysfunction. The internal homogeneity as indicated by Spearman’s correlation coefficient (r) between component scores and the global PSQI score was 0.15–0.81. All the correlation coefficients were significant (*p* < 0.001) (Table 3). The groups identified as normal sleep and insomnia based on clinical interview differed across the global PSQI score and all the components score except the component for medicine use and daytime dysfunction (Table 4). The ROC curve is shown in Fig. 1. Table 5 shows the results of the ROC curve analysis with sensitivity and specificity for all the global PSQI scores between 0.5 and 16. The sensitivity and specificity of the PSQI at the cut-off score of 5.5 were 82 and 56.2%, respectively.

**Table 3:** Internal consistency and homogeneity of the Pittsburgh Sleep Quality Index (PSQI) scores in community dwelling Ethiopian adults

Components of the PSQI	Component-to- global PSQI score correlations	Cronbach’s Alpha if Component Deleted
PSQI component of sleep quality	.50	.58
PSQI component of sleep latency	.56	.52
PSQI component of sleep duration	.81	.43
PSQI component of sleep efficiency	.81	.45
PSQI component of sleep disturbances	.34	.58
PSQI component of sleep medication	.18	.60
PSQI component of daytime dysfunction	.15	.60

**Table 4:** Discriminative validity: Comparison of the Pittsburgh Sleep Quality Index (PSQI) scores between normal sleepers and insomniacs as determined by clinical interview in community dwelling Ethiopian adults

Components of the PSQI	Mean rank		*p*-value
	Normal sleepers	Primary Insomniacs	
PSQI component of sleep quality	90.88	189.19	<0.01
PSQI component of sleep latency	94.47	187.36	<0.01
PSQI component of sleep duration	131.25	168.61	<0.01
PSQI component of sleep efficiency	131.11	168.68	<0.01
PSQI component of sleep disturbances	130.65	168.92	<0.01
PSQI component of sleep medication	155.00	156.51	0.52
PSQI component of daytime dysfunction	154.40	156.82	0.58
Global PSQI score^a^	4.70 ± 3.46	8.11 ± 2.61	<0.01

^a^Mean ± SD, Independent *t*-test was used for the global PSQI score and Mann Whitney *U* test was applied for component scores

**Table 5:** Sensitivity and specificity of the Pittsburgh Sleep Quality Index at each cut-off score in community dwelling Ethiopian adults

Cut-off Score	Sensitivity	Specificity
0.5	0.99	0.14
1.5	0.99	0.29
2.5	0.97	0.35
3.5	0.95	0.40
4.5	0.91	0.46
5.5	0.82	0.56
6.5	0.73	0.65
7.5	0.61	0.74
8.5	0.53	0.83
9.5	0.41	0.93
10.5	0.12	0.96
11.5	0.05	0.99
12.5	0.02	0.99
14	0.01	1.00
16	0	1.00

Tables 6, 7 and 8 shows the results of the factor analysis. The results of Kaiser-Meyer-Olkin test of sampling adequacy, Bartlett’s test of sphericity, anti-image matrix and determinant show that the sample met the conditions for factor analysis (Table 6) [11, 16]. Kaiser’s criteria (Eigenvalue > 1), Scree plot and parallel analysis identified 3-factor models, while cumulative variance rule (>40%) found 2-factor model for the PSQI with seven components. Kaiser’s criteria (Eigenvalue > 1), Scree plot and parallel analysis identified 2-factor model, while cumulative variance rule (>40%) found 1-factor model for the PSQI with five components (without the PSQI components of medicine use and daytime dysfunction) (Table 6).

**Table 6:** Summary of the sample size adequacy measures and exploratory factor analysis of the Pittsburgh Sleep Quality Index in community dwelling Ethiopian adults

Measures	PSQI (Seven components)	PSQI (Five components)
Kaiser-Meyer-Olkin Test of Sampling Adequacy	0.51	0.52
Bartlett’s test of Sphericity	<0.001	<0.001
Anti-image matrix	0.39–0.67	0.48–0.64
Determinant	0.08	0.09
Number of factors		
Kaiser’s criteria (Eigenvalue > 1)	3	2
Cumulative variance rule (>40%)	2	1
Scree plot	3	2
Parallel analysis	3	2

*PSQI* Pittsburgh sleep quality index

**Table 7:** Factor loadings in exploratory factor analysis of the Pittsburgh Sleep Quality Index in community dwelling Ethiopian adults

Components of the PSQI	PSQI (Seven components)			PSQI (Five components)	
	Factor 1	Factor 2	Factor 3	Factor 1	Factor 2
PSQI component of sleep quality	.88	.05	.08	.88	.05
PSQI component of sleep latency	.87	−.13	.18	.87	−.13
PSQI component of sleep duration	.10	−.96	.01	.10	−.97
PSQI component of sleep efficiency	.09	−.97	.06	.09	−.97
PSQI component of sleep disturbances	.62	−.13	.08	.62	−.12
PSQI component of sleep medication	.16	−.03	.78		
PSQI component of daytime dysfunction	.06	−.03	.81		

Principal component analysis with direct oblimin rotation (Kaiser Normalization) was employed. *PSQI* Pittsburgh sleep quality index

**Table 8:** Summary of the Confirmatory factor analysis of the Pittsburgh Sleep Quality Index in community dwelling Ethiopian adults

Models	GFI	AGFI	CFI	RMSEA	RMR	*χ* ^2^	df	*p*	*χ* ^2^/df	ECVI
Model-A	0.79	0.58	0.32	0.34	0.29	528.06	14	<0.01	37.72	1.79
Model-B	0.98	0.95	0.98	0.06	0.04	22.26	10	0.01	2.23	0.19
Model-C	0.80	0.57	0.35	0.35	0.29	508.91	13	<0.01	39.15	1.74
Model-D	0.74	0.23	0.33	0.56	0.40	490.30	5	<0.01	98.06	1.65
Model-E	0.98	0.91	0.98	0.11	0.05	15.09	3	<0.01	5.031	0.13

Model-A: 1-Factor model of the PSQI with all the seven components; Model-B: 1-Factor model of the PSQI with all the seven components and incorporation of modification index (correlations between error terms); Model-C: 2-Factor model of the PSQI (Factor-1 comprised of SLPQUAL, LATEN, DURAT, HSE, DISTB; Factor-2 comprised of MEDS, DAYDYS); Model-D: 1-Factor model of the PSQI with only five components (without MEDS and DAYDYS); Model-E: 1-Factor model of the PSQI with only five components (without MEDS and DAYDYS) with incorporation of modification index (correlations between error terms)

*GFI* goodness of fit index, *AGFI* adjusted goodness of fit index, *CFI* comparative fit index, *RMSEA* root mean square error of approximation, *RMR* root mean square residual, *ECVI* expected cross-validation index

SLPQUAL: PSQI component of sleep quality, LATEN: PSQI component of sleep latency, DURAT: PSQI component of sleep duration, HSE: PSQI component of sleep efficiency, DISTB: PSQI component of sleep disturbances, MEDS: PSQI component of sleep medication, DAYDYS: PSQI component of daytime dysfunction

The CFA was run on five models of the PSQI (Table 8); Model-A: 1-Factor model of the PSQI with all the seven components; Model-B: 1-Factor model of the PSQI with all the seven components and incorporation of modification index (correlations between error terms); Model-C: 2-Factor model of the PSQI (Factor-1 comprised of the PSQI components for sleep quality, sleep latency, sleep duration, sleep efficiency and sleep disturbances; Factor-2 comprised of the PSQI components for sleep medicine and daytime dysfunction); Model-D: 1-Factor model of the PSQI with only five components (without the PSQI components for sleep medicine and daytime dysfunction); Model-E: 1-Factor model of the PSQI with only five components (without the PSQI components for sleep medicine and daytime dysfunction) with incorporation of modification index (correlations between error terms). None of the models had absolute fit to the data i.e. non-significant *χ* ^2^ p value (Table 8). Three models performed poorly i.e. RMR and RMSEA were higher than the cut-off values, while GFI, AGFI and CFI were lower than the cut-off values. Model-B performed best with highest values for GFI, AGFI and CFI, while it had lowest values for RMSEA, RMR and *χ* ^2^/df.

## Discussion

This is the first study to examine the psychometric and diagnostic validity of a sleep questionnaire tool in any segment of the Ethiopian population. In this study, the PSQI was validated in community dwelling Ethiopian adults using ICSD-R criteria for screening of insomnia. The individual components of the PSQI had floor and ceiling effects but the global PSQI score did not have either of these effects (Table 2). Therefore, item analysis does support validity of the overall score of the scale [14]. One of the few studies that reported about floor and ceiling effects found that floor effects were observed for all the PSQI components except for sleep disturbances (Table 2) in patients of temporomandibular disorders [17].

The internal consistency as assessed in this population of community dwelling Ethiopian adults was moderately adequate. Although, the value of Cronbach’s alpha was low in this study, it may be noted that the tool has never been reported to show a value of this psychometric index within the ideal range i.e., 0.9–0.95. Previous studies have reported Cronbach’s alpha values between 0.58 and 0.83 [5, 6, 17]. However, Rener-Sitar et al. 2014 reported a value of Cronbach alpha (.58) in patients of temporomandibular disorders without complaints of pain [17], which is almost similar to the one found in our study (Table 2). The component-global PSQI score correlation was moderate to strong except for the PSQI component of medicine and daytime dysfunction (Table 2). A recent systematic review concluded that the sleep medicine component has been shown to contribute poorly to construct validity [5]. Additionally, lack of awareness about sleep health in developing societies is common [18]. The lesser awareness might lead to contrived low sensitivity of this component in such societies [4, 10].

The significantly higher values of the global PSQI and component scores (except daytime dysfunction and sleep medicine) among insomniacs establish the diagnostic known-group or discriminative validity of the tool in this population of community dwelling Ethiopian adults. Notably, with regard to discriminative validity a striking similarity was observed with the previous report in African population from Nigeria. The validation of the tool in the Nigerian students found that the global PSQI and component scores (except daytime dysfunction and sleep medicine) were significantly higher among insomniacs [7]. The relatively less contribution of the PSQI component of daytime dysfunction and sleep medicine to internal consistency, homogeneity, and discriminative validity in Afro-Asian populations [4, 7, 10] is interesting and need to be explored further. This may help further increase the utility of the tool in these populations and understanding of sleep health construct in these societies.

The diagnostic validity of the scale against ICSD-R criteria for insomnia in this sample of community dwelling Ethiopian adults was in moderate to adequate range. The AUC of 0.78 (Fig. 1) found in our study was slightly less than the recommended limit value of 0.80 for good diagnostic use [19]. However, it is higher than those reported by other studies with different gold standard and/or concurrent measure in diverse samples [5]. The value was almost similar to that reported in patients with lower back pain; AUC was 0.79 (CI 0.723–0.819; *p* < 0.0001), for identifying insomnia. The concurrent measure employed in that study was sleep diary [8]. However, the value of AUC in our study was higher than that reported in Nigerian students; AUC (0.685), this study employed a measure of concurrent validity that was similar to that used in our study [7]. The cut-off score (5.5) (Table 5 and Fig. 1) for screening insomnia in our study sample of community dwelling Ethiopian adults was higher than that reported in Nigerian students and less than that estimated in patients of post-acute brain injury [7, 9]. The global PSQI score cannot take values in decimals [6], therefore the practical cut-off score in our study was 6-a value similar to that reported in patients of lower back pain for screening insomnia [8]. The sensitivity (82%) and specificity (56.2%) of the PSQI at the cut-off score was comparable to previous studies validating the tool for screening of primary insomnia [7–9]. The results of the EFA were inconclusive, but the outcome of CFA favored unidimensionality of the PSQI in the Ethiopian community adults (Tables 6, 7 and 8). This is similar to some of the previous reports [5, 11, 17], though heterogeneity of the factor structure of the PSQI remains an area of extensive research [5, 7, 11, 16].

The limitations of this study include a modest sample size, and non-application of polysomnography. The gender ratio of the sample was not representative of the general Ethiopian population. Therefore, the results of the study may be gender biased for males. Future work should explore this aspect. However, the merit of the study include concurrent measure of clinical screening by sleep researchers based on ICSD-R, validation in a population having high prevalence of sleep problems which may be related to chat addiction and that does not have access to polysomnography, actigraphy and trained sleep health professionals. The PSQI was found to be of adequate use for screening for insomnia among this sample of community dwelling Ethiopian adults.

## Additional file

Additional file 1: The dataset used in validation of the Pittsburgh Sleep Quality Index in the community dwelling Ethiopian adults. (SAV 22 kb)
